# Use of Artificial Intelligence in Rheumatoid Arthritis: Advancements and Novel Perspectives

**DOI:** 10.3390/jcm15145482

**Published:** 2026-07-13

**Authors:** Rossella Talotta, Felice Sfravara, Filippo Fiorentino, Emmanuele Barberi, Sebastiano Gangemi

**Affiliations:** 1 Rheumatology Unit, Department of Clinical and Experimental Medicine, University of Messina, 98124 Messina, Italy; filippo.fiorentino@studenti.unime.it; 2 Department of Engineering, University of Messina, 98122 Messina, Italy; felice.sfravara@unime.it (F.S.); emmanuele.barberi@unime.it (E.B.); 3 School and Operative Unit of Allergy and Clinical Immunology, Department of Clinical and Experimental Medicine, University of Messina, 98124 Messina, Italy; sebastiano.gangemi@unime.it

**Keywords:** rheumatoid arthritis, artificial intelligence, machine learning, deep learning, autoimmunity, imaging, predictivity, generative artificial intelligence, diagnosis

## Abstract

**Highlights:**

**What are the main findings?**

- RA is a complex disease with many unmet needs.
- AI may improve the management of RA patients by enabling early identification of the disease and stratifying patients based on prognostic factors or treatment response.
- ML models have proven accurate at combining large volumes of clinical and omics data to address some of these aspects.
- DL techniques may be more effective for image interpretation.

**What is the implication of the main finding?**

- The main limitations of AI in clinical practice are small cohorts, poor data generalizability, and the lack of external validation.
- GenAI, Agentic AI, and FL may represent future frontiers for addressing current AI gaps in clinical practice.

**Abstract:**

Rheumatoid arthritis (RA) is a chronic systemic inflammatory disease that can cause severe joint damage and disability. The management of RA patients has evolved significantly over the past few decades due to improved detection of early disease progression and the initiation of targeted advanced treatments. However, several gaps remain, including disease risk assessment, early diagnosis, phenotypic identification, and risk of treatment failure. These gaps could potentially be addressed by the application of artificial intelligence (AI), defined as the ability of a machine to mimic intelligent human behavior, using machine learning and deep learning models. By analyzing various types of data, including clinical, laboratory, and imaging data, omics, demographics, and data from sensor applications or wearable technologies, it may be possible to improve the management of RA patients, as demonstrated by several studies. However, limitations related to interindividual variability, small datasets, study designs, and intrinsic model bias remain, making it difficult to generalize findings to larger cohorts of RA patients. The aim of this narrative review is to discuss the potential uses and limitations of AI-based algorithms in RA patients in light of the most recent scientific evidence.

## 1. Introduction

Rheumatoid arthritis (RA) is a systemic, chronic autoimmune disease characterized by synovial inflammation of diarthrodial joints and complications involving other organs. The disease affects approximately 0.1–0.6% of the global population, with wide variability across continents depending on selection criteria and risk factors [1]. Genetic predisposition (such as carrying the polymorphic allele human leukocyte antigen *HLA-DR4*), female sex, cigarette smoking, chronic periodontitis, inhaled pollution, infections, and dysbiosis are potential triggers of RA [1,2]. RA pathogenesis centers on the invasion of the synovial membrane by immune cells, among which T and B lymphocytes are key players in the immunologic response. In particular, B lymphocytes differentiate into plasma cells and produce several autoantibodies, of which rheumatoid factor (RF) and anti-citrullinated peptide antibodies (ACPAs) are the most pathognomonic. The molecular mechanisms underlying this scenario have been greatly elucidated through various experiments. In brief, HLA encoded by polymorphic variants, including *HLA-DR4*, would be more likely to present peptides that undergo post-translational modifications, such as citrullination, leading to the activation of T lymphocytes and B lymphocytes [3]. Cigarette smoking and periodontal infections caused by *Porphyromonas gingivalis* may both contribute to increased citrullination or carbamylation of self-proteins, raising the likelihood of RA development [3]. Antigen-presenting cells (APCs) that present post-translationally modified peptides to naive T cells mainly drive their differentiation into T helper (Th)1, Th2, and Th17 cells. Th17 cells are of particular importance, as they can mediate several pathogenic processes, including neutrophil chemotaxis, synovial cell proliferation, macrophage activation, and cartilage and bone damage [4]. Extravasation of T cells into the synovial membrane and subsequent activation of B cells lead to RA synovitis. Inflammation of the synovial membrane in RA is a cornerstone process whose pathogenetic steps include: (1) in situ formation of germinal center B cells and autoantibody production; (2) stimulation of macrophage-like synoviocytes (MLSs) to secrete pro-inflammatory cytokines, including interleukin-1 (IL-1), IL-6, and tumor necrosis factor (TNF)-α; (3) fibroblast-like synoviocyte (FLS) proliferation; and (4) release of matrix metalloproteinases (MMPs) [4]. These events lead to the formation of a synovial pannus, which ultimately induces cartilage loss and bone erosion. Patients may carry autoantibodies for several years before developing clinical manifestations [3]. Symptoms usually begin around 40 years of age and include joint pain, morning stiffness, and loss of function, typically affecting multiple joints in the hands and feet in a symmetrical, centripetally migrating pattern [5]. If not promptly recognized and treated, chronic synovitis can lead to irreversible joint deformities, significantly affecting patients’ function and quality of life.

Patients may also experience inflammation of tendons and bursae, as well as general malaise, fever, and weight loss. In the most severe cases, the disease can be complicated by damage to organs such as the eyes, lungs, skin, and heart [5,6]. In particular, patients with RA have a twofold increased risk of ischemic heart disease, especially in cases of long-standing, seropositive disease [7].

Thanks to the most recent classification criteria for RA that identify early forms of the disease, the widespread adoption in clinical practice of treat-to-target and tight control strategies, and the availability of many therapeutic options, the prognosis of patients has greatly improved compared to previous decades [8,9,10,11]. Currently, RA can be managed with monotherapy or combinations of conventional synthetic (cs), biologic (b), and targeted synthetic (ts) disease-modifying anti-rheumatic drugs (DMARDs), the latter two of which have been specifically developed to halt the immunopathogenic scenario that characterizes the disease [12]. However, despite improvements in the therapeutic algorithm for RA, a significant number of patients remain refractory [13]. Indeed, the long-term evolution of RA is extremely variable, with some patients achieving good control of disease activity and others developing joint deformities and disability [14]. Treatment delay, higher disease activity, and female sex have been identified as independent predictors of refractory disease [15].

Such gaps in disease risk assessment, early diagnosis, phenotypic identification, and refractory case detection could potentially be addressed by applying artificial intelligence (AI), defined as the ability of a machine to mimic intelligent human behavior, through machine learning (ML) and deep learning (DL) models [16,17]. In clinical practice, AI can integrate and process clinical, laboratory, and instrumental data, assisting clinicians in early or difficult diagnosis, treatment selection, and patient follow-up [18]. Several lines of research have shown that applying AI to RA may have a significant impact on early diagnosis, prognosis, and patient risk stratification [19]. Limitations to this approach are mainly due to a lack of validation or reliance on small datasets, which may lead to misinterpretation of results and poor generalizability [20]. Future developments toward more robust, large-scale, validated, and more interpretable algorithms are expected to enable real integration of AI into clinical practice, contributing to the transition toward precision medicine in rheumatology [18].

The aim of this paper is to review the current scientific evidence regarding the advantages and disadvantages of AI application to the diagnosis, prognosis, treatment, and stratification of patients with RA, and to provide a new perspective on the potential use of AI in this field.

## 2. Materials and Methods

**Figure 1:**
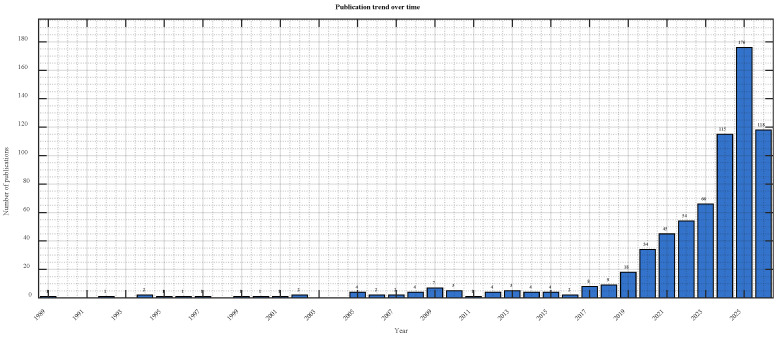
Paper publication trend over time by the use of “rheumatoid arthritis” together with “artificial intelligence” as keywords in Scopus database.

For this narrative review, we consulted Scopus and PubMed, two leading international databases that collect high-impact scientific literature. Using the combined keywords “rheumatoid arthritis” and “artificial intelligence,” a literature search was conducted to identify relevant studies addressing the application of computational methods in this clinical domain. The total scientific output indexed since 1983, as of 30 June 2026, includes 659 documents in Scopus (Figure 1) and 783 documents in PubMed. This reflects a marked increase in scientific production over the last decade, particularly after 2010, in parallel with the emergence and rapid development of DL approaches in biomedical research.

To provide a structured overview of this rapidly evolving field, the literature was selected and organized to develop a structured conceptual framework for the analysis of AI applications in RA. Preference was given to systematic reviews or meta-analyses, clinical studies, phase I to IV trials, comparative studies, controlled studies, observational studies, research support articles, and randomized controlled trials (RCTs) published in the last 20 years, written in English, and preferably indexed in both databases. This search strategy, completed on 30 June 2026, led to the final selection of 130 articles published from 1983 to date, 49 of which are clinical studies that specifically address the use of AI in RA.

These studies were not intended to represent an exhaustive systematic collection of all available evidence, but rather a curated set of works suitable for the construction of a structured taxonomy of the field. The selected studies were analyzed and classified according to three dimensions: (i) type of data used, (ii) computational task addressed, and (iii) category of AI algorithm employed. Table 1 summarizes the classification framework adopted in this review.

## 3. Results

### 3.1. Artificial Intelligence: From Conceptualization to Practical Application

The field of AI originated in the 1950s and focuses on the study and development of intelligent systems—that is, systems capable of performing tasks that, to an external observer, would appear to require human-like behavior, such as learning from experience, reasoning, and decision-making [21]. Unlike traditional computer programming, which relies on explicit instructions and rigidly defined rules, AI algorithms are designed to operate even in previously unseen scenarios, adapting their behavior based on data and acquired experience [22].

This adaptability is enabled through the use of ML techniques, in which models are trained on datasets according to different paradigms, including supervised, unsupervised, and semi-supervised learning. In the first case, the model learns from labeled data, that is, data associated with correct outputs; in the second, it autonomously identifies hidden structures and patterns within the data; in the third, it combines elements of both approaches. More recent methodologies, such as reinforcement learning, further extend this framework by allowing an agent to learn through interaction with an environment, receiving feedback in the form of rewards or penalties [23].

From a modeling perspective, AI encompasses a wide range of techniques, including statistical models, decision tree-based algorithms, kernel methods, and, more recently, deep artificial neural networks, or DL, which are loosely inspired by the functioning of the human brain. These models have proven particularly effective in complex tasks such as image recognition, natural language processing (NLP), and time series forecasting [22,24].

AI applications are now widespread and span multiple domains, including recommendation systems used in digital platforms [25], autonomous vehicles [26], speech [27] and visual recognition [28], as well as decision-support tools in industrial and financial contexts [29].

In the medical domain, these technologies are emerging as promising tools for clinical data analysis, diagnostic support, and treatment personalization, although significant challenges remain in terms of reliability, interpretability, and integration into real-world settings.

### 3.2. Artificial Intelligence in Healthcare

The use of AI in the medical field has become increasingly important in recent years. Thanks to its ability to synthesize input from structured and unstructured sources, accelerate the diagnostic process, improve accuracy, and reduce costs—especially in prevention—AI could radically transform healthcare [30]. The impact of AI in healthcare is already particularly evident in medical imaging and diagnostics, drug discovery and research, virtual patient care, robotic surgery, rehabilitation, genomics, and precision medicine [30,31]. Among the main branches of AI, ML is a fundamental tool capable of analyzing large amounts of data and developing predictive models that support clinical decision-making, contributing to the evolution toward more predictive and personalized medicine [32]. DL, a subset of ML, enables the automatic identification of complex patterns directly from raw data and has proven particularly effective in analyzing medical images and recognizing clinically relevant features [32]. More recently, generative artificial intelligence (GenAI), based on advanced DL models, has been able to learn from heterogeneous datasets, interpret complex clinical information, and simulate decision-making processes [33]. However, a meta-analysis of studies validating GenAI models for diagnostic tasks found a diagnostic accuracy of 52.1%, with no significant difference in performance between AI and non-specialist doctors, although the diagnostic accuracy of AI was significantly lower than that of specialist doctors [34].

### 3.3. Artificial Intelligence in Rheumatic Diseases

The need for early diagnosis in rheumatic diseases and the potential to assist non-rheumatologists in the diagnostic process using AI-based methods dates back to the 1980s, when an AI-based system called AI/RHEUM was developed to detect 26 rheumatic diseases by incorporating formal criteria [35]. The system was initially tested on 384 clinical cases, achieving an accuracy of 94%. A subsequent Japanese study involving 59 patients with connective tissue diseases (CTDs) confirmed agreement between the AI tool and traditional methods for making a correct diagnosis in 92% of cases, with a sensitivity of 90% and a specificity of 96% [36].

Nowadays, AI applications are emerging as increasingly relevant tools in rheumatology, helping clinicians with imaging interpretation, disease monitoring, and therapeutic decision support. The availability of big data collected from billions of patients during the examination of medical records, adverse drug reactions, genomics, social media, or clinical trials, and the possibility of processing and interpreting such data through AI software, has attracted the attention of international societies such as the European League Against Rheumatism (EULAR). In 2020, EULAR formulated three overarching principles and ten points addressing ethical issues and methods of data collection and analysis [37]. Recognizing that “big data provides unprecedented opportunities to deliver transformative discoveries in rheumatic musculoskeletal disease (RMD) research and practice”, EULAR strongly promotes interdisciplinary collaboration, shared open data platforms, and training in big data methods for scientists. However, the experts also suggest caution regarding AI methods, which should be compared to identify the best-performing ones, benchmarked, and validated. Indeed, a systematic literature review conducted in 2019 on the use of AI in RMD big data concluded that both big data sources and methodologies were heterogeneous [38]. Most of the data were clinical, in contrast to other diseases where data were obtained from omics and medical imaging. The definition of big data was often conflicting among the 55 included studies, and there was confusion regarding volume, multidimensionality, and dynamic interconnections. Although ML was the most commonly used technique (97% of cases), 5–10% of the analyzed studies did not clearly mention the algorithm used.

In subsequent years, the body of scientific evidence on the potential application of AI in rheumatology has been enriched by further studies aimed at assessing disease activity, enabling earlier diagnosis, improving discrimination in imaging interpretation, and predicting treatment response, as shown in Table 2. ML and DL techniques have been tested for various indications in numerous rheumatic diseases, including inflammatory arthritis, CTDs, vasculitis, gout, osteoarthritis (OA), and osteoporosis (OP).

Overall, the results of these studies show that AI methods can predict unfavorable treatment responses and identify risk factors for treatment failure in seronegative arthritis such as psoriatic arthritis (PsA) [39,40]; discriminate radiological features to assist in the diagnosis of axial spondyloarthritis (axSpA) [41,42,43]; identify disease subtypes and complications and predict worse outcomes in systemic lupus erythematosus (SLE) [44,45,46,47]; support diagnosis based on histopathology data in lupus nephritis (LN) and Sjögren’s syndrome (SS) [48,49,50]; instrumentally detect interstitial lung disease (ILD) even in asymptomatic CTD patients [51]; discriminate instrumental signs of large- and medium-vessel vasculitis [52,53]; predict recurrence of gout [54]; facilitate early diagnosis of OA and support OA patient self-management in training and rehabilitation programs [55,56,57,58]; and identify patients at risk of OP [59]. In studies comparing the performance of AI models with human expertise, the accuracy of such algorithms was comparable to that of experts and even superior to that of junior physicians [41,42,50].

However, although promising, AI cannot entirely replace physicians’ opinions. AI models may perform very well in experimental studies, but their relevance in clinical practice is still limited [60]. The complexity of many rheumatic diseases requires multimodal and integrative models based on large, standardized datasets [18]. The lack of these data, the difficulty of integrating them into clinical practice, and challenges in model interpretability currently limit the applicability of AI in clinical settings. At present, AI remains a useful tool to support clinical reasoning but cannot substitute for medical expertise.

**Table 2:** Studies on the application of AI in multiple rheumatic diseases.

Rheumatic Disorder	Study Design	Patients	Endpoint	Results	Author, Date, Reference
PsA	Multicenter, prospective, noninterventional study	1278 eligible PsA patients treated with or scheduled to be treated with SEC from the AQUILA dataset	- Prediction of LDA and high HRQOL response at 16 weeks using binary ML; - Identification of predictors and quantification of their impact for individual patients using an XAI model	- Patient global assessment, physician global assessment, previous use of bDMARDs, TJC, and age emerging as the main LDA predictors; - Impact of disease, BDI, height, TJC, and BMI predicted to be associated with high HRQOL	Vodenčarević et al., 2025 [39]
SpA	Multicenter, prospective study collecting data from the BIOBADASER registry	969 axial SpA patients who started treatment with a TNFi	- Identification of predictors of response to TNFi using a combination of statistical and AI methods (Anaxomics AI Data Science software) to extract relevant features from large datasets	- Female sex, age at diagnosis and at treatment initiation, and comorbidities predicted to be associated with an unfavorable response to TNFi	Fernández-Carballido et al., 2023 [40]
	Multicenter, prospective, noninterventional study	683 eligible axSpA patients treated with or scheduled to be treated with SEC from the AQUILA dataset	- Prediction of LDA and high HRQOL response at 16 weeks using binary ML; - Identification of predictors and quantification of their impact for individual patients using an XAI model	- BASDAI, previous use of bDMARDs, CRP values, ASAS HI, and height identified as predictors of LDA; - ASAS HI, BDI, BMI, height, and age predicted to be associated with high HRQOL	Vodenčarević et al., 2025 [39]
	Prospective multicenter study extrapolating MRI data from the DESIR and ASAS cohorts	256 patients from the DESIR cohort with inflammatory back pain lasting more than 3 months and less than 3 years, of whom 27% met the ASAS criteria for SpA; 47 patients from the ASAS cohort, with 19% meeting the ASAS criteria for active sacroiliitis, used for external validation	Comparison of sensitivity, specificity, accuracy, Matthews correlation coefficient, and AUC of a DL model (Mask-RCNN) trained to detect active sacroiliitis on MRI according to the ASAS definition, compared to expert majority opinion	Similar performance of the DL model and experts in identifying BMO in sacroiliac joints and detecting sacroiliitis according to the ASAS definition	Bordner et al., 2023 [41]
	Retrospective multicenter study using MRI scan datasets from the Genodisc, Oxford Whole Spine, PREVENT, and MEASURE-1 studies	686 axSpA patients enrolled in the MEASURE-1 and PREVENT trials	- Assessment of the accuracy of ML models (SpineNet software), with and without manual segmentation, for automated scoring of vertebral BMO on MRI	- Both models showing performance comparable to expert readers in detecting BMO; - Applying ML models without manual segmentation performed better than using an intermediate manual segmentation step	Jamaludin et al., 2025 [42]
	Retrospective multicenter study collecting conventional pelvis X-ray images from the PROOF, GESPIC, OptiRef, and DAMACT datasets	2170 adult axSpA patients from the PROOF dataset, with 1483 X-rays used as training data; 525 axSpA patients from the GESPIC study and 361 axSpA patients from the OptiRef study, with 436 and 340 radiographs, respectively, used for inference analysis; plus 178 patients (89 with axSpA and 74 without SpA) from the DAMACT dataset used for inference	Comparison of AUC, accuracy, sensitivity, and specificity for detection and progression prediction of sacroiliitis between two neural networks: one anatomy-centered on the sacroiliac joints and the other on the full X-ray image	- Similar learning performance for both the standard and anatomy-centered models; - The anatomy-centered model shows higher consistency across datasets, as well as higher AUC scores, accuracy, sensitivity, and specificity compared to the comparator; - Ability of anatomy-centered models to identify patients progressing to radiographic sacroiliitis within 2 years	Dorfner et al., 2024, [43]
SLE	Multicenter study	446 cases, of which 382 allocated to the training and validation cohort (36 patients with cutaneous manifestations of SLE or other cutaneous diseases and 18 healthy controls) and 64 allocated to the external test cohort	Development and testing of an MMDLS for predicting cutaneous lupus subtypes	- Better performance of the MMDLS in diagnosing 13 skin conditions compared with single or dual models; - Increased diagnostic accuracy of junior dermatologists when the MMDLS is consulted	Li et al., 2024 [44]
	Multicenter study and AI-enhanced meta-analysis	12,850 patients with SLE from registry cohorts in Asia, Europe, North America, and Africa	Evaluation of the diagnostic and prognostic significance of NLR and PLR in SLE using an XGBoost ML model, guided by the Transparent Reporting of a Multivariable Prediction Model for Individual Prognosis or Diagnosis-AI, with SHAP, integrating traditional clinical biomarkers and multi-omics data	- Superior diagnostic accuracy of NLR compared with PLR for predicting active SLE, although with significant ethnic variation; - Higher risk of LN, CV events, or mortality associated with high NLR and PLR; - Improved accuracy in predicting active SLE when NLR and PLR are integrated into the AI model	Ali et al., 2025 [45]
	Observational study	395 SLE patients requiring first hospitalization from the MIMIC-IV database and 100 critically ill patients with SLE allocated to the validation cohort	Development and validation of two predictive models: a traditional model based on logistic regression and an ML model using a stacking ensemble approach for assessing mortality risk in critically ill SLE patients	- AUC above 0.8 reported for both models; - The ML model outperformed logistic regression in terms of precision and specificity; - SHAP analysis made it possible to show the contribution of individual variables	Chen et al., 2025 [46]
	Systematic review	Over 800 patients, including NPSLE, SLE without neuropsychiatric involvement and healthy individuals	Evaluation of the performance of AI-based methods (ML or DL) in detecting NPSLE by integrating neuroimaging, CSF, or serum biomarkers	- Most studies relying on neuroimaging rather than biomarkers; - High performance of AI-based methods (pooled AUC 0.86; accuracy 0.87), but significant between-study heterogeneity that influenced sensitivity analyses; - No significant differences between ML and DL algorithms; - Lack of standardization, external validation, and explainable methods in most studies	Nouroozi et al., 2026 [47]
LN	Monocenter retrospective study	58 GN patients	Prediction of LN outcome and remission achievement by testing an AI-based neural network integrating laboratory and histopathological data	- Best performance of a multilayer perceptron with 40 neurons in the first hidden layer and 45 neurons in the second hidden layer (accuracy of 91.67%) with 100% precision for predicting complete LN remission	Stojanowski et al., 2022 [48]
	Multicenter cohort study	31,670 patients with chronic kidney disease across 6 medical centers	Evaluation of the performance of AI software in diagnosing glomerular disease by analysis of kidney TEM images	- Accuracy of TEM-AID in identifying glomerulonephritis subtypes, demonstrating high internal diagnostic performance and consistent external validation across five test sets; - Superior diagnostic performance of TEM-AID compared with pathologists	Ma et al., 2025 [49]
SS	Multicenter cohort study	100 patients, of whom 36 were used for training and internal validation datasets and 64 for the external test dataset	Assessment of the accuracy of CTG-PAM, based on graph theory, in scoring and diagnosing SS by analyzing cells and tissues in salivary gland biopsies	- High accuracy and better performance of CTG-PAM compared to traditional DL methods in diagnosing SS; - CTG-PAM’s diagnostic accuracy similar to that of expert pathologists and higher than that of junior pathologists	Wu et al., 2024 [50]
CTDs	Case series	59 Japanese patients	Evaluation of the accuracy of diagnosing a CTD by the AI/RHEUM tool compared to the diagnosis made by a rheumatologist	- Full or partial agreement between the AI tool and traditional methods of diagnosing CTDs (92%), with a sensitivity of 90% and specificity of 96%	Porter et al., 1988 [36]
	Cross-sectional study	67 patients with CTD and early ILD development, including 21 with SSc, 23 with IIM, 9 with SS, 7 with SLE, 5 with MCTD, and 2 with RA	Accuracy of AI-based methods (AlqpHRTC) in diagnosing early lung involvement in CTDs (asymptomatic versus symptomatic) by analyzing HRCT datasets and combining these data with clinical and functional information	- Increased detection of high-attenuation lung volume and reticulations in symptomatic patients by AlqpHRTC, but not of ground-glass opacities or honeycombing; - High reliability of AlqpHRTC in detecting ILD features in asymptomatic patients	Hoffmann et al., 2025 [51]
Vasculitis	Retrospective multicenter study	137 GCA patients	Detection of the halo sign in CDU of temporal arteries using a DL approach	- Excellent accuracy in standardized images; - Identification of pathological signs in 90% of cases from non-standardized images; - Thrombus may increase the risk of bias	Roncato et al., 2020 [52]
	Retrospective monocenter study	1474 KD patients subdivided into training and validation cohorts at an 80/20 ratio	Development and validation of a DL AI model to detect coronary artery lesions	- Best performance achieved with a decision tree model based on 24 demographic, laboratory, and clinical features	Yang et al., 2025 [53]
	Systematic review and meta-analysis	103,882 participants, of whom 12,541 were diagnosed with KD	Evaluation of the accuracy of ML methods to distinguish KD from other febrile conditions	- Twenty-nine studies included, of which 20 used ML; - The ability to identify KD showed a sensitivity of 0.91 and a specificity of 0.86	Zhu et al., 2024 [61]
Gout	Real-world, retrospective, prospective, multicenter cohort study	6526 hospitalized gout patients, of whom 4074 were used to develop the prediction model, while 1746, 360, and 346 patients were allocated to groups for internal validation, external validation, and the prospective set, respectively	Prediction of gout recurrence through the construction and validation of a multidimensional AI model that takes into account diverse data categories, including comorbidities	- 3744 models screened, of which the IterImp_MM_FS_GB model was found to be the most effective for predicting gout recurrence; - 20 key predictors identified by SHAP analysis, including serum urate levels, neutrophil count, and tophi in patients with multiple comorbidities such as neoplasm and cardiovascular diseases	Li et al., 2025 [54]
	Observational study	6648 gout patients allocated to GIMS and 1651 gout patients allocated to EMRS	Comparison between the GIMS and EMRS cohorts in terms of kidney function preservation and maintenance of the serum urate target	- Reduced incidence of chronic kidney disease stage ≥ 3 and higher achievement of the target serum urate level in the GIMS group compared to the EMRS group	Qi et al., 2025 [62]
OA	Single-blinded RCT	82 knee OA patients	Development of an interactive mobile application that uses AI to provide personalized physical exercise programs to patients based on knee OA severity	- Higher accuracy in performing three prescribed exercises in patients using the mobile application than in controls; - Improvements in quality of life, function, and satisfaction in patients assigned to the mobile application group compared to controls	Thiengwittayaporn et al. 2023 [55]
	Monocenter validation study	5849 Thailand patients of whom 3455 had knee OA	Validation of DL models to predict early diagnosis of knee OA by processing textual data such as clinicians’ notes on patient symptoms	- Best performance achieved by the BiLSTM model, which improved further after applying a WOMAC-based processing approach; - Better performance of this model compared to other methods that use imaging or laboratory data	Thanyakunsajja et al., 2025 [56]
	Phase 2, double-blinded, observational study	50 patients with knee OA	Comparison between an AI-generated (GPT-4) self-management guide for OA and recommendations created by clinicians	Better performance of GPT-4 compared to clinicians in content generation speed, accuracy, personalization, safety, and comprehensiveness	Du et al., 2025 [57]
	Cross-sectional study	40 patients with knee OA	Comparing personalized rehabilitation programs generated by AI (ChatGPT-4.0 and Gemini Advanced) with physiotherapists’ consensus programs	- Greater agreement between ChatGPT-4.0 and physiotherapists’ consensus programs compared with Gemini Advanced; - Comparable or better performance of ChatGPT-4.0 than Gemini Advanced in most phases; - Limitations in exercise specificity, including frequency, sets, and progression criteria	Gürses et al., 2025 [58]
	Retrospective analysis study	5966 knee radiographic images from the Osteoarthritis Initiative dataset used for model development, and 3392 knee radiographic images from the Multicenter Osteoarthritis Study dataset used for validation	Development of an ML-assisted method to predict KLG progression over 4–5 years in patients starting from a KLG score of 0, 1, or 2, integrating additional predictors such as demographics, comorbidities, history of meniscectomy, gait speed, WOMAC scores, and X-ray findings	- The model demonstrated good accuracy in predicting the progression of knee OA over 4–5 years	Lee et al., 2025 [63]
	Retrospective analysis study	600 patients with knee OA enrolled in the Foundation for the National Institutes of Health Osteoarthritis Biomarkers Project, 297 of whom were classified as pain progressors according to the WOMAC scale during a 24- to 48-month follow-up period	Development of a nomogram model based on radiomics data (Neusoft Discovery) and clinical characteristics to predict pain progression	- Excellent predictive capability and accuracy in predicting pain progression using X-ray radiomics-based nomograms	Sun et al., 2026 [64]
OP	RCT	40,658 participants aged 40 years or older who underwent chest radiography without a history of DXA examination	Identification of individuals at high risk of OP using an AI model applied to chest radiographs	- 4912 participants classified as at high risk of OP by the AI model; - high proportion of patients with OP detected by DXA in this selected population; - increased odds ratio of OP in screened subjects who did not meet the formal criteria for DXA compared with those who did	Lin et al., 2024 [59]
Miscellanea of inflammatory rheumatic diseases	Prospective, multicenter, open-label crossover RCT	600 patients from 3 rheumatology centers	Assessment and comparison of the diagnostic accuracy of the AI-based tools Ada and Rheport versus a diagnosis of inflammatory rheumatic disease made by rheumatologists	- Overall diagnostic accuracies of 52%, 63%, and 58%, respectively, for Rheport and Ada’s top 1 and top 5 disease suggestions; - Heterogeneous accuracy of Ada in making individual diagnoses, with its best performance in detecting RA compared with other diseases; - Poor agreement between Rheport and Ada’s top 1 and top 5 disease suggestions	Knitza et al., 2024 [65]

Abbreviations: ASAS: assessment of spondyloarthritis international society; ASAS HI: assessment of spondyloarthritis international society health index; AUC: area under the ROC curve; axSpA: axial spondyloarthritis; BASDAI: Bath ankylosing spondylitis disease activity index; BDI: Beck depression inventory; bDMARDs: biologic disease-modifying anti-rheumatic drugs; BiLSTM: bidirectional long short-term memory; BMI: body mass index; BMO: bone marrow edema; CDU: color Doppler ultrasound; CRP: C-reactive protein; CSF: cerebrospinal fluid; CTG-PAM: cell-tissue-graph-based pathological image analysis model; CTDs: connective tissue diseases; CV: cardiovascular; DL: deep learning; DXA: dual-energy X-ray absorptiometry; EMRS: electronic medical records system; GCA: giant cell arteritis; GIMS: gout intelligent management system; KD: Kawasaki disease; KLG: Kellgren–Lawrence grade; HRQOL: health-related quality of life; IIM: idiopathic inflammatory myopathy; ILD: interstitial lung disease; LDA: low disease activity; LN: lupus nephritis; MCTD: mixed connective tissue disease; ML: machine learning; MMDLS: multimodal DL system; MRI: magnetic resonance imaging; NLR: neutrophil-to-lymphocyte ratio; NPSLE: neuropsychiatric systemic lupus erythematosus; OA: osteoarthritis; OP: osteoporosis; PLR: platelet-to-lymphocyte ratio; PSA: psoriatic arthritis; RA: rheumatoid arthritis; RCT: randomized controlled trial; SEC: secukinumab; SHAP: Shapley additive explanation; SLE: systemic lupus erythematosus; SS: Sjogren’s syndrome; SSc: systemic sclerosis; SpA: spondyloarthritis; TEM: transmission electron microscopy; TJC: tender joint count; TNFi: tumor necrosis factor inhibitor; WOMAC: Western Ontario and McMaster Universities Arthritis Index; XAI: explainable artificial intelligence.

### 3.4. Artificial Intelligence and Rheumatoid Arthritis: The State of the Art

Both ML and DL models have been applied to RA [19]. Their major advantage is the ability to combine heterogeneous data, including medical records, imaging, and genomics, which can be especially useful for imaging interpretation, early diagnosis, disease subtype classification, prediction of treatment response, and personalized therapies. The following sections analyze these aspects according to the different RA domains where AI has been applied. The results of the main studies are shown in Table 3.

#### 3.4.1. Imaging

The vast majority of studies have investigated the accuracy of AI in image interpretation. The diagnosis of RA indeed relies on imaging techniques, including ultrasound (US), X-rays, and magnetic resonance imaging (MRI). AI applications in RA imaging have shown promising results, particularly in automating the identification of inflammatory and structural lesions. Thanks to the ability of DL to process large amounts of unstructured data, such AI methods could compensate for current pitfalls in traditional image interpretation by improving accuracy, efficiency, and consistency. Consequently, in recent years, several studies have developed and tested DL models to improve diagnostic accuracy and standardize image interpretation while reducing dependence on operator experience. US, plain radiography, and MRI images of hands and wrists, and, in a few cases, feet, have been used as input sources for various DL models, including convolutional neural networks (CNN), U-shaped convolutional neural networks (U-Net), Random Forest, and extreme gradient boosting (XGBoost) [66].

US is particularly important in RA not only for diagnosis through the detection of synovitis but also for monitoring disease progression. This imaging technique offers many advantages, as it is safe, noninvasive, cost-effective, and allows real-time evaluation in both grayscale and power Doppler (PD) modalities [67]. The main limitation of US is its dependence on operator expertise, but this may be addressed by applying AI models. Some research has shown that, when applied to the automatic evaluation of US images, AI achieves accuracy similar to that of rheumatologists. In 2019, Andersen et al. reported for the first time that an AI model based on neural networks could distinguish between healthy and affected joints in RA patients according to the OMERACT–EULAR Synovitis Scoring (OESS) system, achieving accuracies of 86.4% and 86.9% compared with an expert rheumatologist [68]. Furthermore, when AI models are trained to recognize synovial hypertrophy and joint effusion, they may achieve a sensitivity of 100% and a Dice coefficient of 84%, respectively [69]. In this regard, Chang et al. developed a Self-Attention U-Net-based model for automatic recognition of wrist synovial hypertrophy and joint effusion in patients with RA [69]. The model demonstrated strong segmentation capability for inflamed synovial structures, highlighting the potential of AI in automated US image analysis. CNN models have also been shown to be effective in scoring disease activity ultrasonographically by processing both static and dynamic images, in grayscale and PD modes. These data, reported in He’s study [70], increasingly support the application of AI to routine musculoskeletal US, in which the complexity of anatomical structures makes it difficult to rely solely on single static grayscale or PD images.

Radiography has traditionally been the gold standard for detecting joint space narrowing and bone erosion. However, conventional scoring systems are highly prone to inaccuracies. Before 2019, ML models were trained to recognize radiographic features corresponding to various stages of RA. After 2019, interest shifted primarily to DL models, which have been trained to recognize, for example, synovitis [71] and bone erosions [72], as well as ankylosis and subluxation in wrist and hand radiographs [20]. The results of a study published in 2019, which used a combination of ML and DL to identify and quantify joint damage according to the Sharp/van der Heijde method and compared these findings with clinicians’ expertise, reported exact agreement rates of 49.3–65.4% for joint space narrowing and 70.6–74.1% for bone erosion [73]. Importantly, the authors observed underdiagnosis of erosions and overdiagnosis of joint space narrowing, which may be attributed to the small dataset and the difficult evaluation of intercarpal joints by the models. These limitations could be addressed by processing radiographic images from the contralateral hand or by comparing the same group of joints, for example metacarpophalangeal or proximal interphalangeal joints. By applying a DL model that combined and integrated such information to 226 hand X-ray images from 40 patients with RA, Miyama et al. obtained detection rates for all target joints of 98.0% for erosion and 97.3% for joint space narrowing [72]. This approach enabled automated assessment of joint erosions, producing results superior to those of orthopedic surgeons. Similarly, Okita et al. reported promising results using a DL model for the automatic identification of atlantoaxial subluxation in cervical radiographs of 906 patients with RA. The model identified atlantoaxial subluxation with a sensitivity of 0.97 and a specificity of 0.57, showing minimal differences compared with clinicians [20].

MRI is considered the gold standard for visualizing RA synovitis, bone edema, and cartilage loss. However, it also has limitations, including examination duration, cost, and variability in image interpretation between operators. AI has shown nearly identical capabilities in quantifying bone edema and tenosynovitis in wrist MRI compared to visual assessments [74]. DL models have also been trained to automatically classify RA severity, demonstrating potential in assessing disease progression [19]. MRI is also an important area of application for AI in early RA. Aizenberg et al. developed an automated system for quantifying bone marrow edema at the wrist using MRI in patients with early arthritis [75]. The study demonstrated the possibility of automatically identifying and quantifying subclinical inflammatory changes, supporting the potential use of AI in early diagnosis and monitoring of disease activity. Similarly, Gaj et al. proposed an automated synovitis segmentation system using MRI, demonstrating the feasibility of DL techniques for objective quantification of synovial inflammation in patients with RA [76].

Overall, these studies highlight how ML and DL models applied to different imaging techniques can improve automated detection of inflammatory manifestations and structural damage in RA, opening important perspectives for more standardized, rapid, and reproducible assessment of the disease. However, DL techniques still face certain limitations that may lead to image misinterpretation. This may be due to the erroneous acquisition of spurious correlations or the identification of irrelevant features, such as imaging artifacts. Other gaps include scale variation, boundary ambiguity, and the difficulty of modeling long-range dependencies. To overcome these limitations, explainability techniques or transformer models could be used [66].

#### 3.4.2. Early Diagnosis

Early diagnosis of RA is a crucial clinical challenge, as the early stages of the disease are often characterized by nonspecific symptoms and significant biological heterogeneity [11]. In this context, AI has effectively improved diagnostic sensitivity through the analysis of multimodal data, including clinical, serological, and imaging parameters. Specifically, supervised ML models, such as Random Forests and support vector machines, have been used to combine clinical and autoantibody variables to identify patients with early arthritis who are at risk of developing RA [77]. In parallel, DL approaches, particularly CNN, can directly analyze US and MRI images, enabling automatic recognition of subclinical inflammation patterns, such as synovitis and other inflammatory imaging features [69]. The integration of these models into multimodal approaches also improves early risk stratification, distinguishing transient forms from those likely to progress to persistent and erosive RA [78].

Predicting the progression of seronegative undifferentiated arthritis to RA can be challenging, as the sensitivity of current RA classification criteria is less than 20% in the absence of autoantibodies. Building on this observation, Fujii et al. used various ML models based on clinical and laboratory data to assess their predictive capacity for disease progression in two cohorts of patients with undifferentiated arthritis [79]. Among the tested models, the Feed-forward neural network (FNN) proved to be the most accurate, making it a strong candidate for diagnostic decision-making based on routine clinical data. More recently, Rahimi et al. tested five ML algorithms to identify key factors associated with early diagnosis of RA in primary care [77]. By analyzing clinical and laboratory variables, the authors identified predictive patterns useful for recognizing high-risk individuals in the early stages of the disease, highlighting the potential of AI as a decision-support tool in daily clinical practice. These included autoantibodies, tender and swollen joint counts, gastrointestinal disorders, fatigue, age, and hearing impairments.

Furthermore, AI-assisted analysis of metabolomic patterns may differentiate RA from other painful joint conditions. In a multicenter study by Tang et al., five ML algorithms were applied to determine the plasma metabolomic profile of RA patients compared with OA patients and healthy controls. The results identified six metabolites that could be considered candidate diagnostic biomarkers of RA, independently of autoantibody status [80]. These included N-acetyl-L-methionine, imidazole acetic acid, ergothioneine, 1-methylnicotinamide, 2-keto-3-deoxy-D-gluconic acid, and dehydroepiandrosterone sulfate. Although the applied model did not account for disease activity or concomitant medications, it proved to be stable and reproducible across different samples and analytical methods.

The use of AI for early RA diagnosis has also been extended to clinical data from electronic health records (EHRs). Maarseveen et al. developed and validated an ML pipeline for the automatic identification of patients with RA from EHRs [81]. The model integrated structured and unstructured data, including diagnosis codes, medication prescriptions, and free text, enabling accurate classification of patients with RA within the healthcare population.

Regarding MRI, Aizenberg et al. developed an automated system to quantify wrist tenosynovitis in patients with early arthritis [75]. Using automated MRI image analysis techniques, the model identified and quantified tendon inflammatory changes associated with early-stage disease, supporting the potential use of AI in the early diagnosis of RA. Similarly, Li et al. applied a DL model based on extremity MRI images to classify and predict RA, demonstrating high discriminatory power in distinguishing RA patients from control subjects [82].

Overall, these studies show that integrating clinical, laboratory, and imaging data using ML and DL techniques can promote earlier and more accurate identification of RA, helping optimize diagnostic and therapeutic resources for patients. The main limitations lie primarily in the need to use sensitive data, which raises security and privacy concerns. In addition, the poor explainability of some models makes them unsuitable for routine clinical practice, and there are significant challenges in obtaining and standardizing historical data [83]. Data quality, overfitting, and integration into the clinical setting are further obstacles to the diffusion of ML models into clinical practice. Model optimization and simplification, with particular regard to compatibility with healthcare systems, as well as collaboration across disciplines, may represent strategies to solve this issue [83].

#### 3.4.3. Identification of Clinical Phenotypes

The identification of RA clinical and biological phenotypes is an area of growing interest for the application of AI, aiming to improve patient stratification and support personalized medicine approaches. By integrating clinical, genetic, metabolomic, and electronic data, ML models can identify complex patterns that are difficult to detect using traditional statistical methods.

RA is a complex disease with a heterogeneous clinical presentation and outcomes. Patients can display diversity in autoantibody titers, genetic signatures, gut microbiota composition, activated immunologic pathways, synovial histology, comorbidities, long-term outcomes, and treatment response [84,85]. While this latter aspect has been the topic of many studies and will be discussed in more detail in the next section, studies using AI to stratify patient endotypes are more limited.

A way to differentiate subsets of RA patients using AI models may rely on the analysis and processing of electronic medical records (EMRs). Pham et al. used ML models to generate an RA case definition algorithm based on structured and unstructured data from primary care electronic data [86]. By integrating diagnostic codes, drug prescriptions, and free-text analysis, the model enabled accurate classification of patients with RA, contributing to the identification of clinical subgroups with different disease patterns. Another study investigated four ML approaches to analyze and cluster comorbidities from inpatient and outpatient medical records of 1643 participants with and without RA from eight Minnesota counties [87]. The authors found significant associations among mental and behavioral comorbidities, as well as among cardiovascular risk factors and diseases in both RA patients and controls; these associations depended on both age and sex. When considering comorbidities clustered within each cohort, patients with RA did not demonstrate important differences compared with patients without RA. However, missing comorbidities, different methodologies, lack of information on disease severity, seropositivity, and concomitant treatment may have biased these findings.

Indeed, although real-world datasets provide a valuable source of clinical information that could be decisive in RA diagnosis and phenotype stratification, several current limitations constrain this approach. Among them, missing data—especially regarding prognostic outcomes—and the inclusion of potential confounders must be kept in mind. Thanks to the ability to elaborate on and leverage unstructured clinical information from physician notes, NLP may overcome these biases [88]. For instance, in the multicenter study by Román Ivorra et al., the application of an NLP model to 64,241,683 EHRs allowed the identification of 13,958 patients with RA, of whom 5.1% had ILD. The model also allowed the identification of clinical features associated with the RA-ILD phenotype, including older age, greater disease severity, and comorbidities [89].

Another intriguing key for RA patient stratification could be the integration of multi-omics layers. Synovial biopsies and blood samples may offer valuable information on genomics, transcriptomics, proteomics, and metabolomics [85]. AI models capable of deciphering such molecular signatures may open new scenarios for precision medicine. In a study, ML algorithms examining transcriptomic data from bioptic samples successfully identified patients with RA and renal fibrosis and traced a specific transcriptomic profile [90]. In another ML-assisted transcriptomic analysis study on synovial membrane samples from RA patients and subjects with pigmented villonodular synovitis (PVNS), Heng et al. identified 107 differentially expressed genes and three hub genes (*PLIN*, *PPAP2A*, and *TYROBP*) that distinguished RA from PVNS [91]. Interestingly, these three genes showed a significant association with distinct immune cell profiles, namely infiltration of macrophages, plasma cells, dendritic cells, and monocytes in PVNS, and natural killer cells, neutrophils, macrophages in RA.

In another study, Orange et al. applied ML algorithms to histological and transcriptomic data obtained from synovial membrane samples from 123 RA patients and six OA patients, identifying three distinct molecular endophenotypes defined by different immunological pathways and cellular profiles [92]. By training a histology ML model on these subtypes, the authors found that the high-inflammatory subtype was defined by the following histologic features: binucleated plasma cells, the percentage of plasma cells, and Russell bodies. Thus, this approach helped distinguish between individuals with RA and OA and identify RA patients with more aggressive disease who may benefit from specific therapeutic interventions.

Overall, these studies demonstrate that applying ML to multi-omics, genetic, and clinical data can improve the phenotypic characterization of RA, promote more precise patient stratification, and open new perspectives for personalized therapeutic approaches. However, limitations remain, including privacy concerns, poor model explainability, and bias due to missing data or selected populations, which currently restrict the broader adoption of these techniques in routine clinical practice.

#### 3.4.4. Treatment Response and Personalized Therapies

The heterogeneity of treatment response to csDMARDs, bDMARDs, and tsDMARDs remains a major challenge in RA. If not contraindicated, methotrexate (MTX) remains the anchor drug for the treatment of the disease [93]. bDMARDs and tsDMARDs can be considered for RA patients with previous failure of MTX or other csDMARDs, or for those with poor prognostic factors. Among them, anti-TNF agents, or TNF inhibitors (TNFi), are the first bDMARD option because of clinicians’ extensive experience and their more favorable cost-effectiveness profile. However, up to 40% of TNFi-treated patients discontinue treatment because of primary non-response, loss of response, or intolerance [94]. Secondary therapeutic strategies may include switching to another TNFi or to a drug with a different mechanism of action, including tsDMARDs. Current international guidelines do not clearly specify the use of a particular drug class, leaving the choice to clinicians, who should consider potential contraindications. Consequently, the most common clinical approach is trial and error, which can delay optimal treatment selection and increase the risk of disease progression and permanent structural damage [14].

Predicting treatment response could be a key application of AI in RA, aiming to support increasingly personalized therapeutic strategies. Some of the most widely used models are Random Forests, support vector machines, and GBoost. These models can analyze nonlinear relationships and integrate multiple clinical variables, including age, sex, disease duration, disease activity, laboratory biomarkers (erythrocyte sedimentation rate or ESR, C-reactive protein or CRP, RF, ACPA), and imaging (US, MRI) [95].

In this context, Lim et al. developed an ML model that integrates genetic and clinical data to identify a predictive signature of MTX response in patients with RA [96]. The study showed that specific combinations of clinical variables and genetic polymorphisms can define distinct patient subgroups, enabling better phenotypic stratification and more accurate prediction of therapeutic response. The early identification of patients at risk of inadequate response to conventional treatments was further explored by Duquesne et al., who applied ML techniques to define a clinical profile associated with failure to respond to MTX [97]. By integrating clinical and laboratory variables, their model recognized specific patterns associated with insufficient treatment response, supporting the possibility of early referral of these patients to alternative or more aggressive therapeutic strategies.

Several studies also report the accuracy of AI methods in predicting response to bDMARDs or tsDMARDs.

For instance, Salehi et al. developed ML models to predict response to bDMARDs based exclusively on routine clinical data collected at baseline [98]. Using several supervised algorithms, including XGBoost, Random Forest, and AdaBoost, the authors achieved high predictive performance for both initial and sustained responses, highlighting the potential for using readily available data in clinical practice to guide treatment decisions. Specifically, the AdaBoost model showed strong performance in estimating remission probabilities, with milder baseline disease activity, including better clinimetric scores and low levels of ESR and CRP, considered candidate predictors. However, the lack of integration of genetic or imaging data, the response criteria chosen, and the selected populations to which the models were applied were major gaps in this study, which relied solely on clinical data.

Bouget et al. applied ML algorithms to predict response to TNFi in patients with RA using data from the ESPOIR and ABIRISK cohorts [95]. By integrating clinical, biological, and demographic variables, their model identified patterns associated with therapeutic response to TNFi, demonstrating the potential of AI to support more targeted and personalized treatment decisions. Similarly, Chen et al. developed a ML framework based on a Stacked-Ensemble pipeline to predict response to TNFi treatment in bionaive RA patients by integrating routinely collected clinical data. The model showed good discriminatory capacity for the early identification of patients who did not respond to treatment according to the EULAR criteria [99].

The combination of genetic and clinical data may improve the robustness of the prediction models. In this regard, Guan et al. developed ML models to predict response to TNFi by combining genetic biomarkers known to be associated with either RA risk or TNFi response and clinical parameters [100]. The study showed that adding genetic information significantly improves the predictive power of the models compared with using clinical data alone.

An even more advanced approach was proposed by Tao et al., who combined multi-omics data and ML algorithms to predict clinical response to adalimumab and etanercept in patients with RA [101]. The study analyzed transcriptomic, proteomic, and clinical data to identify specific molecular signatures associated with therapeutic response. The findings showed distinct transcription signatures in RA patients who responded to etanercept or adalimumab, which were consistent across different immune cell types. Despite limitations due to the small sample size and number of dropouts, this study may pave the way for future research on personalized medicine approaches.

In one study, ML models were applied to estimate the rate of disease remission in patients treated with the IL-6 receptor inhibitor tocilizumab (TCZ), comparing RCT and real-world cohorts [102]. Compared with logistic regression, ML models improved the accuracy of remission prediction in a real-world patient cohort after 24 weeks of TCZ monotherapy.

More recent research has highlighted the importance of applying AI to predict response or adverse events in RA patients undergoing treatment with Janus kinase inhibitors (JAKi). For example, we report the work of Lee et al., who aimed to develop and validate ML models based on baseline clinical variables to predict response to tofacitinib or baricitinib [103]. The study, conducted on 264 RA subjects from the KOBIO registry with at least 12 months of follow-up, identified distinct clinical and laboratory factors associated with response to either baricitinib or tofacitinib, including inflammatory markers, lipid profile, and type of joint involvement.

Concerning JAKi safety, Hetland et al. analyzed data from 19 clinical trials of RA patients treated with tofacitinib using ML algorithms (support vector machines with linear kernel, Random Forest, XGBoost, and boosted trees) to decipher baseline factors associated with serious infections [104]. The models did not meet the threshold for accurate prediction, with an area under the receiver operating characteristic (AUROC) < 0.85. Identified risk factors included older age, glucocorticoid co-treatment, and previous infections. In another retrospective cohort study, a Random Survival Forest ML algorithm was used to predict the risk of venous thromboembolism events in older RA patients receiving bDMARDs and tsDMARDs [105]. Varicose veins, number of inpatient and outpatient visits, and access to emergency care were estimated as predictors. The model showed good accuracy compared to RegCox analysis.

Overall, these studies highlight that ML is a promising tool for predicting therapeutic response in RA, enabling more accurate patient stratification and contributing to the development of personalized therapeutic strategies, especially when based on the integration of clinical, biological, and genetic data.

Although the results are promising, limited external validation and model heterogeneity currently restrict their clinical use [106,107]. Missing and inconsistent data, along with variable inclusion criteria and assessment of treatment response, are potentially surmountable obstacles. One solution could be multidisciplinary patient management and educational programs that train healthcare professionals to establish uniform, unambiguous management practices across multiple research centers.

#### 3.4.5. Follow-Up

Clinical follow-up of patients with RA may also benefit from AI-assisted programs that incorporate clinimetric, laboratory, and imaging data to predict flares, disability risk, remission or treatment discontinuation. However, studies in this field remain limited.

There is evidence that autoantibody status may predict a more aggressive disease course. In a retrospective cohort study of RA outpatients, ML models (support vector machines, Random Forest, XGBoost, Adaptive Boost, and k-nearest neighbors) predicted worse clinimetric outcomes in patients having antinuclear antibodies (ANAs), especially when associated with FR and ACPA positivity [108].

Combining imaging information may enhance the predictive performance of models, as shown in the paper by Matsuo et al. [109]. In this work, the authors reported that applying ML algorithms (Random Forest and XGBoost) to 73 baseline RA US, laboratory, and clinical features may predict disease relapse or remission. XGBoost showed the best performance, identifying ten predictors, including wrist and metatarsophalangeal superb microvascular imaging scores.

Another study applied GBoost tree models to clinical data from 5481 RA patients followed for at least 5 years to predict the risk of surgery and associated risk factors [110]. The findings suggest that the type and number of medications used may be a predictor of future surgical procedures. Protracted use of glucocorticoids and csDMARDs, rather than escalation to bDMARDs, may predict the need for surgery, highlighting that the major risk may be undertreatment. Conversely, the study did not find any association with demographic features or comorbidities, nor did it discriminate between major and minor surgery procedures, which may be due to the homogeneity and small size of the cohort.

In contrast to the risk of disability and the need for surgery, there is the rare possibility that arthritis may spontaneously resolve. The use of AI-based methods may help identify such cases and reduce overtreatment, which carries a risk of potential adverse events. This may be achieved through the extrapolation of a specific immunologic profile that may predict a more benign course. In a study by Yeo et al. [111], the authors applied a ML approach to identify molecular signatures in synovial biopsies of RA patients and early arthritis patients followed for 18 months. The chemokines CXCL4 and CXCL7 emerged as the most significant predictors of RA development.

Finally, an interesting way to continuously assess RA patients’ disease activity may rely on the use of digital health technologies, such as smartphones and wearables. In a 14-day observational study conducted on 30 moderate-to-severe RA patients and 30 controls, sensor data recorded from daily iPhone-guided tests and an Apple smartwatch were analyzed through an ML framework to distinguish between patients and controls and to evaluate RA disease activity using the Routine Assessment of Patient Index Data 3 (RAPID-3) score [112]. The authors observed that this approach could more faithfully reflect oscillations in RA severity that may be missed during scheduled periodic visits. Remote monitoring of patients through wearable devices and digital technology may represent another innovative way to more effectively control the course of RA, enabling timely treatment adjustment.

A study conducted at Tongji Hospital evaluated an AI technology integrated into a smartphone app that collected patient-entered clinical information from individuals with RA, who also received personalized education and interventions [113]. Compared with the control group, RA patients managed with AI technology achieved better clinical responses and a higher rate of medication adherence at 6 months, and reported high satisfaction with the platform.

**Table 3:** Overview of the main studies dealing with the application of AI in RA.

Domain	Study Design	Patients	Intervention	Results	Author, Date, Reference
Imaging	Retrospective observational study	1694 US images obtained from 40 RA patients with long-standing (n.20) and untreated (n.20) disease	Application of two CNNs with different basic architectures (VGG-16 and Inception-v3) to classify hand and wrist joints as either healthy or diseased and to score images according to the OESS	- Good accuracy in distinguishing healthy from diseased scores compared with an expert rheumatologist (86.4% and 86.9%, respectively); - Accuracy of 75.0% for the Inception-v3 architecture model in four-class OESS	Andersen et al., 2019 [68]
	Retrospective study	Dataset of musculoskeletal US images of the wrist from patients with suspected or known joint pathologies	Development of a Self-Attention U-Net-based DL model for synovitis segmentation and identification	- High accuracy in detecting synovial hypertrophy and effusion; - Improved performance compared with traditional U-Net models; - Greater ability to capture spatial patterns	Chang et al., 2024 [69]
	Retrospective study	1244 US hand and wrist images obtained from 156 RA patients	Validation of four DL models based on a ResNet-type architecture to detect and score synovitis according to the OESS in static grayscale, dynamic grayscale, static PD, and dynamic PD, and comparison with a team of radiologists with varying levels of experience	- Dynamic PD, static grayscale, dynamic grayscale and static PD models emerged as the best-performing models for scores of 0/1/2/3, respectively; - Comparable results between DL models and experienced radiologists on a per-image basis; - Better performance of dynamic DL models than static models in most scoring processes and higher accuracy compared to radiologists	He et al., 2024 [70]
	Retrospective study	216 hand radiographs of 108 patients with RA	Validation of a deep CNN for radiographic evaluation of joint space narrowing and bone erosion according to the Sharp/van der Heijde method	- Accuracy of 49.3–65.4% for joint space narrowing and 70.6–74.1% for erosion; - Discrete agreement between AI scores and clinicians’ judgment (correlation coefficient = 0.72–0.88 for joint space narrowing and 0.54–0.75 for erosion)	Hirano et al., 2019 [73]
	Retrospective study	216 patients with RA undergoing hand and wrist radiographs	Development of a DL system that integrates contextual information from multiple joints to assess bone damage	- High accuracy in erosion assessment; - Improved accuracy compared with context-free models; - Ability to reproduce standard radiographic scores; - Reduced interobserver variability	Miyama et al., 2022 [72]
	Retrospective study	326 RA patients who underwent cervical radiographs	Development of a DL model for automatic detection and classification of atlantoaxial subluxation on radiographic imaging in patients with RA	- High accuracy in identifying subluxation; - Good concordance with specialist assessment; -Potential support for screening and monitoring of RA cervical complications	Okita et al., 2023 [20]
	Retrospective study	Dataset of MRI images obtained from 26 RA patients	Development of a DL algorithm for automatic segmentation of synovitis on MRI	- Good accuracy in segmenting synovitis; - Reduced analysis time compared to manual assessment; - Potential improvement in reproducibility and standardization	Gaj S et al., 2020 [76]
Imaging and early diagnosis	Retrospective feasibility study	30 patients with early arthritis who underwent wrist MRI	Development of an automated algorithm for quantifying tenosynovitis on MRI in patients with early arthritis	- Good correlation with standard semiquantitative scoring, high reproducibility, and potential for objective assessment of early periarticular inflammation	Aizenberg et al., 2019 [74]
	Retrospective feasibility study	30 patients with early arthritis who underwent wrist MRI	Development of an automated algorithm for quantifying bone marrow edema on wrist MRI in patients with early arthritis	- Good feasibility and correlation with standard semiquantitative assessments; - Potential for objective and reproducible quantification of subclinical inflammation	Aizenberg et al., 2018 [75]
Early diagnosis	Retrospective observational study	2151 participants with recent-onset arthritis or clinically suspected arthritis (including healthy individuals)	Development of five ML models using symptoms, demographic data, and laboratory markers as input	- Moderate ability to predict the development of RA; - Ability to detect early signs of disease by MRI	Li et al., 2024 [82]
	Retrospective multicenter cohort study with external validation	350 patients with seronegative undifferentiated arthritis from 2 independent cohorts (210 from the KURAMA cohort used for training and 140 from the ANSWER cohort used for validation)	Development and external validation of a DL FNN integrating clinical and laboratory variables to predict progression to RA; model interpretability assessed using SHAP analysis.	- Excellent predictive performance of the FNN model (AUC of 0.924 in the training cohort and 0.777 in the external validation cohort); - MMP-3 emerged as the most influential predictor, followed by inflammatory and clinical variables	Fujii et al., 2025 [79]
	Retrospective, single-center, observational, analytical study	377 participants, divided into diagnosed RA patients (54%) and symptomatic non-RA controls (46%)	Development of five ML models using symptoms, demographic data, and laboratory markers as inputs	- Strong ability to identify the most predictive features for early RA and potential support for early referral to primary care	Rahimi et al., 2026 [77]
	Multicenter cross-sectional, case–control study	2863 subjects, including patients with RA (with clinical subgroups such as seronegative RA), OA, and healthy controls, recruited from seven independent cohorts and five clinical centers	- Development and validation of ML models based on targeted metabolomics analysis of plasma and serum samples to identify metabolic biomarkers; - Application of logistic regression, LASSO, Random Forest, SVM, and XGBoost to classify RA versus controls and RA versus OA	- Identification of 6 key-metabolites with diagnostic value, including L-phenylalanine, L-tryptophan, L-tyrosine, arachidonic acid, linoleic acid, and lactic acid; - Good discriminatory performance of ML models	Tang et al., 2025 [80]
	Multicenter retrospective observational study	7771 patients from two independent cohorts including RA and non-RA subjects	Development of an ML model using NLP techniques, applied to structured and unstructured data from EMRs to identify patients with RA	- High accuracy in identifying patients with RA and good generalizability, with the model transferable between different centers	Maarseveen et al., 2020 [81]
Clinical phenotype identification	Multicenter retrospective observational study	5555 primary care patient records, including both RA patients and non-RA controls	Development of a case definition for RA using ML on EHRs, integrating structured information and free text with variable selection and supervised model training (Decision Tree, Random Forest, and XGBoost)	- High accuracy in identifying patients with RA and supporting the definition of computational clinical phenotypes	Pham et al., 2024 [86]
	Retrospective, population-based study	Inpatient and outpatient medical records of 1643 participants with and without RA from 8 Minnesota counties	Clustering comorbidities in RA patients using unsupervised ML methods (hierarchical clustering, factor analysis, k-means clustering, and network analysis)	- Significant associations among mental and behavioral comorbidities, as well as among cardiovascular risk factors and diseases, in both RA patients and controls; - Associations between mental and behavioral comorbidities and younger age, and between dementia and older age; - Gender-specific comorbidities clustered together; - Numerous differences in comorbidity clustering between RA and non-RA cohorts, but these differences were minimized when considering comorbidities clustered together within each cohort	Crowson et al., [87]
	Multicenter observational case–control, retrospective study	3,176,165 patients from 9 hospitals, of whom 3958 patients had RA and 5.1% of RA patients additionally had ILD	Study of the prevalence of ILD among RA patients and the characteristics associated with this phenotype versus non-ILD RA patients through the application of NLP to unstructured clinical information from EHRs and its standardization into SNOMED CT terminology	- Precision of the NLP model of 79.4% and 76.4% for ILD and RA, respectively; - Advanced age, infections, malignancies, higher inflammatory burden, pharmacological prescription, and cardiovascular disease among risk factors of RA-associated ILD; - High in-hospital mortality and death in RA patients with ILD	Román Ivorra et al., 2024 [89]
	Retrospective transcriptomic analysis study	- Four datasets from the GEO database, encompassing gene expression profiles from 25 synovial membrane samples from RA patients and 19 from healthy subjects, and 215 samples of renal fibrosis versus 124 controls	Evaluation of transcriptomic signatures in RA and renal fibrosis using a combination of ML algorithms (LASSO and Random Forest) and bioinformatics analysis	- *BIRC3* and *PSMB9* identified as hub differentially expressed genes in RA and renal fibrosis; - AUC of 0.829 through 10-fold cross-validation of the ML model	Qiu et al., 2025 [90]
	Retrospective transcriptomic analysis study	GSE3698 dataset from the GEO database including 18 RA samples and 11 PVNS samples	Evaluation of transcriptomic signatures and potential therapeutic targets of common genes between RA and PVNS, using a combination of ML methods (LASSO and Random Forest)	- 107 differentially expressed genes in RA and PVNS, with identification of 3 hub genes (*PLIN*, *PPAP2A*, and *TYROBP*); - Association of the 3 hub genes with 28 infiltrating immune cell types; - Good diagnostic performance of the model	Heng et al., 2023 [91]
	Translational observational study	129 synovial tissue samples from 123 RA patients and 6 OA controls	Development of an ML model integrating synovial histology, RNA sequencing, and cellular profiling to identify molecular disease subtypes	- Identification of three distinct synovial molecular endotypes characterized by different inflammatory pathways and cellular composition; - Association of a high-inflammatory subtype with three plasma cell features; - Association of a low-inflammatory subtype with expression of fibroid genes and neuronal genes	Orange et al., 2018 [92]
Treatment response prediction	Retrospective, observational study	349 patients treated with MTX, classified as responders or non-responders according to DAS28	Development of an ML model based on the integration of genetic (whole exome sequencing) and clinical data with biologically guided feature selection	- Good predictive performance and potential for application in therapy personalization	Lim et al., 2022 [96]
	Multicenter observational study	870 observations from three independent cohorts (ESPOIR, Leiden EAC, tREACH) of patients treated with MTX	Development of ML models (logistic regression, Random Forest, gradient boosting—LightGBM/CatBoost) using routine clinical and biological data to predict response to MTX at 9 months; Automatic variable selection and interpretation with SHAP used to identify predictive biomarkers	- Moderate predictive ability for response to MTX; - Identification of a clinically interpretable biomarker (lymphocytosis) associated with non-response	Duquesne et al., 2023 [97]
	Retrospective observational study	1223 RA patients from German and Austrian cohorts treated with various bDMARDs, for whom 6- and 12-month follow-up data were available	Development of supervised ML models (XGBoost, AdaBoost, Random Forest, SVM, KNN) using baseline clinical data to predict initial and sustained response to bDMARDs; Nested cross-validation and SHAP used to identify the most relevant variables	- Moderate predictive ability for response to bDMARDs (AUC approximately 0.70–0.85); - Identification of relevant clinical variables, such as age, sex, disease duration, DAS28 scores, ESR, CRP, RF, and ACPA, associated with therapeutic efficacy	Salehi et al., 2024 [98]
	Multicenter observational study	1166 patients with RA treated with anti-TNF agents	Development of supervised ML models to predict response to TNFi using clinical, demographic, and biological variables	- Moderate ability to predict response to TNFi; - Identification of specific clinical and biological variables associated with therapeutic outcomes; - Model robustness confirmed by validation in an independent cohort	Bouget et al., 2022 [95]
	Retrospective observational study	425 RA patients	Development of a Stacked-Ensemble ML model using EHRs data to predict TNFi treatment efficacy	- Good accuracy of the ML framework model in identifying responders versus non-responders according to the EULAR criteria	Chen et al., 2022 [99]
	Retrospective observational study	2700 RA patients treated with TNFi	Development of ML models, including Random Forest, LASSO, and other linear and nonlinear approaches, to predict response to TNFi by integrating clinical and genetic (SNP) data. The model evaluated both continuous outcomes (DAS28 variation) and responder versus non-responder classification	- Improvement in predictive performance with the addition of genetic data (increase in AUC from approximately 0.63 to 0.67); - Superior performance of nonlinear models, such as Random Forest; - Moderate overall predictive ability	Guan et al., 2019 [100]
	Prospective observational study	80 patients with RA who were candidates for TNFi (adalimumab or etanercept)	Development and validation of a Random Forest ML model integrating multi-omics data, including gene expression and DNA methylation from PBMCs, monocytes, and CD4+ T cells, to predict response to TNFi before therapy initiation, with validation in a follow-up study involving a therapeutic switch	- High accuracy of multi-omics models in predicting response to TNFi before treatment initiation; - Identification of distinct molecular signatures between responders and non-responders to either adalimumab or etanercept	Tao et al., 2021 [101]
	Retrospective study	452 RA patients from the Corrona RA registry treated with TCZ monotherapy and 853 matched patients from 4 RCTs	Comparison of the performance of logistic regression and Random Forest models in predicting remission rates under both controlled and real-life conditions	- Remission reported in consistent percentages of RA patients in both RCTs and real-world evidence; - Better discriminatory performance with the application of ML algorithms	Johansson et al., 2021 [102]
	Retrospective multicenter study	264 patients with moderate-to-severe RA from the KOBIO registry and Asan Medical Centers	Training and validation of an XGBoost model to predict response to a 6-month treatment with JAKi	- Remission observed in 65% of patients on tofacitinib and 70% on baricitinib; - High accuracy of the ML model for predicting response to either tofacitinib or baricitinib (80% and 88%, respectively); - Lipid profile, inflammatory markers, and inflamed joint patterns identified as key predictive factors for the response to tofacitinib; - Patient global assessment, joint swelling, and concomitant hydroxychloroquine treatment emerging as key predictive factors for the response to baricitinib	Lee et al., 2025 [103]
	Retrospective multicenter study	8404 RA patients treated with tofacitinib from 19 clinical trials	Prediction of serious infections through the application of statistical and ML methods (logistic regression, support vector machines with linear kernel, Random Forest, XGBoost, and boosted trees)	- Failure of the model to meet the threshold for accurate prediction (AUROC < 0.85); - Older age, glucocorticoid co-treatment, and previous infections identified as risk factors	Hetland et al., 2024 [104]
Follow-up	Retrospective study	210 RA patients from the KURAMA cohort followed up for 2 years	Prediction of RA relapse through the comparative use of three ML classifiers (Logistic Regression, Random Forest, and XGBoost) examining 73 US, laboratory, and clinical features	- Best performance of the XGBoost classifier compared with the other two (AUC = 0.747); - Identification of 10 features that may predict relapse, including wrist or metatarsophalangeal superb microvascular imaging scores	Matsuo et al., 2022 [109]
	Retrospective single-center study	5481 RA patients followed for 5 years	Application of GBoost decision tree models to predict risk of surgery, identify risk factors, and determine type of procedure	- AUC of 0.90 for the model predicting use of surgery and 0.58 for the model predicting type of surgery; - No significant demographic or comorbidity difference between patients who did and did not have surgery; - Prescription of NSAIDs more common among patients who did have surgery; - csDMARD and corticosteroid use having the greatest influence on the model predicting the use of surgery	Baxter et al., 2024 [110]
	Prospective cohort study	48 patients subdivided into early RA, established RA, resolved RA, or uninflamed, undergoing synovial biopsy and an 18-month follow-up	Application of GMLVQ to discriminate among 117 cytokines and related molecule expressions	Significantly increased expression of CXCL4 and CXCL7 in patients with early RA compared with those with resolving arthritis or established disease	Yeo et al., 2015 [111]
	Prospective observational study	30 patients with moderate-to-severe RA and 30 matched healthy controls	Evaluation of the performance of digital technology integrating ML, PROs, and digital health data (iPhone-guided tests and sensor data passively recorded from an Apple smartwatch) in remote disease activity monitoring	- Improved detection of RA severity using sensor-based data compared with PROs alone; - Good reliability in continuously assessing RA status with a combination of the two modalities	Creagh et al., 2024 [112]
	Single-center, multicampus, real-world observational study	341 RA patients with a 6-month follow-up allocated to the intervention or control group	Development of an AI-assisted platform for recording joint symptoms, fatigue, medication adherence, laboratory tests, and emotional status to enhance treatment compliance and remote patient monitoring	- Significant decrease in DAS28 and disability scores in the intervention group compared with the control group; - Higher medication adherence in the intervention group; - Better satisfaction with the AI-based technology platform compared with standard care	Zhang et al., 2026 [113]

Abbreviations: ACPA: anti-citrullinated peptide antibodies; AUC: area under the ROC curve; AUROC: area under the receiver operating characteristic curve; bDMARDS: biologic disease-modifying anti-rheumatic drugs; CNNs: convolute neural networks; CRP: C-reactive protein; DAS28: disease activity score on 28 joints; DL: deep learning; EHRs: electronic health records; EMRs: electronic medical records; ESR: erythrocyte sedimentation rate; EULAR: European League Against Rheumatism; FNN: Feed-forward neural network; GEO: Gene Expression Omnibus; GMLVQ: generalized matrix relevance; ILD: interstitial lung disease; LASSO: least absolute shrinkage and selection operator; JAKi: Janus kinase inhibitors; ML: machine learning; MRI: magnetic resonance imaging; MTX: methotrexate; NLP: natural language processing; NSAIDs: non-steroidal anti-inflammatory drugs; OA: osteoarthritis; OESS: OMERACT–EULAR Synovitis Scoring system; PBMCs: peripheral blood mononuclear cells; PROs: patient-reported outcomes; PVNS: pigmented villonodular synovitis; RA: rheumatoid arthritis; RF: rheumatoid factor; SHAP: Shapley additive explanation; SNP: single nucleotide polymorphism; TCZ: tocilizumab; TNFi: tumor necrosis factor inhibitors; US: ultrasound; XGBoost: Extreme Gradient Boosting.

## 4. Discussion

The application of AI models in rheumatology is continuously expanding due to comparable or even superior accuracy in addressing many unmet needs, including early diagnosis, patient stratification, and interpretation of imaging and histopathology. Clinicians may rely on AI for differential diagnosis of diseases with overlapping symptoms or heterogeneous courses. AI can integrate complex patterns from clinical, imaging, and laboratory data to identify combinations of variables that are difficult to recognize with traditional approaches, thereby increasing diagnostic accuracy, especially in the early stages of disease [18,56,59]. The use of AI models based on imaging, biomarkers, and longitudinal data may predict disease progression, the risk of developing structural damage, the likelihood of flare, and response to treatment [18,41,42,43,51,64]. These models can assist physicians with prognosis and early risk stratification, which is essential for timely interventions. AI may also help identify patient subgroups (endotypes) and, through multimodal approaches, predict drug response and the risk of therapeutic failure, thereby guiding the choice among csDMARDs, biological drugs, and targeted synthetic therapies [18,40,44,47]. Additionally, AI may be useful for dynamic and personalized follow-up by integrating continuous longitudinal data (clinical visits, serial imaging, biomarkers) with digital or wearable devices and apps that record electronic patient-reported outcome (ePRO) scores to identify early disease flares or loss of therapeutic response [18].

RA is undoubtedly one of the most extensively studied rheumatic disorders, with numerous prominent data sources available. This abundance of data facilitates the development and validation of AI models to assist clinicians across various disease domains [114].

In recent years, the use of AI in RA has grown rapidly and progressively, involving many areas of clinical practice and translational research [115]. Current evidence suggests that ML and DL algorithms can significantly improve early diagnosis, phenotypic characterization, imaging assessment, and prediction of therapeutic response. The integration of clinical, laboratory, genetic, multi-omics, and radiological data has enabled the development of increasingly sophisticated predictive models, opening significant prospects for precision medicine in rheumatology.

**Figure 2:**
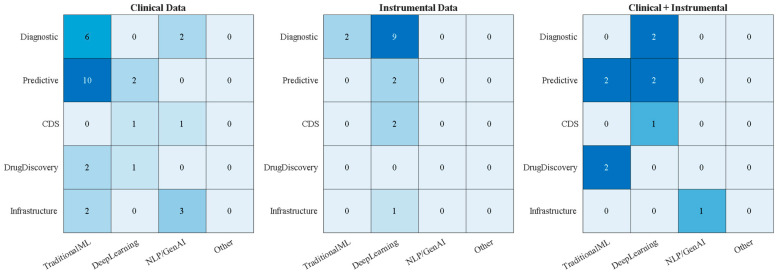
Areas of investigation of AI models in RA. The distribution of studies on RA and AI is shown by data type, task, and AI algorithm. Color intensity represents the number of articles (N), using a unified scale across panels. To enable consistent comparison, a unified color scale was applied across all heatmaps, where color intensity corresponds to the number of articles within each category combination. The analysis is stratified into three panels representing studies based on clinical data, instrumental (imaging/sensor-based) data, and combined clinical plus instrumental data. Each panel reports the frequency of studies across five task categories (diagnostic, predictive, clinical decision support and monitoring, drug discovery and molecular modeling, and healthcare system and infrastructure) and four AI algorithm classes (traditional ML, DL, NLP/generative AI, and other computational approaches). This visualization enables the identification of methodological trends and gaps in the literature, highlighting the predominance of specific AI approaches within each data modality and task domain, as well as the limited representation of certain categories across the current evidence base. Abbreviations: GenAI: generative artificial intelligence; ML: machine learning; NPL: natural language processing.

The AI methodologies described in the studies differ substantially in their underlying principles and fields of application. Their selection largely depends on the nature of the available data and the clinical or research objective to be addressed (Figure 2).

Traditional ML algorithms are particularly suitable for structured datasets, such as clinical, laboratory, genetic, or biomarker variables. These methods typically rely on manually selected features and are effective even with relatively limited sample sizes, making them well suited for predictive modeling and clinical decision-making tasks [116,117]. Among them, NLP techniques enable the extraction and analysis of information contained in unstructured clinical text, including EHRs, medical reports, and scientific documents. However, their performance depends strongly on the quality and representativeness of the selected features.

On the other hand, DL models are based on multilayer neural network architectures that can learn complex patterns directly from high-dimensional data. In RA, these approaches have been predominantly applied to imaging modalities such as radiographs, US, and other instrumental data, where they can automatically extract relevant features without the need for manual feature engineering. This ability makes them particularly effective for diagnostic tasks, although their performance is often dependent on large, annotated datasets and domain-specific training [118,119,120].

From a task perspective, diagnostic applications represented the largest proportion of studies, followed by healthcare system and infrastructure applications, and predictive modeling. This distribution highlights a predominant focus on disease identification and classification tasks within the current AI landscape in RA. In terms of data modality, clinical datasets were the most frequently used, either in isolation or in combination with instrumental data. Instrumental data were primarily associated with imaging-based diagnostic tasks [20,69]. Regarding AI methodologies, traditional ML remained the most widely adopted approach, particularly in predictive and diagnostic modeling using structured clinical variables [81,100,101,106].

DL approaches were mainly applied to instrumental imaging data [20,68,69,70,71,72,73], while NLP methods were predominantly used in healthcare system applications, including clinical documentation analysis [81].

Overall, these findings highlight a clear stratification between data modality and AI methodology, with imaging data strongly associated with DL approaches and structured clinical data predominantly analyzed using traditional ML techniques.

One of the main strengths identified in the analyzed studies is AI’s ability to manage large volumes of heterogeneous, multidimensional data and identify patterns that are difficult to detect with traditional statistical methods [18]. Different ML approaches (neural networks, boosted trees, elastic nets, etc.) that integrate EHRs, imaging, and omics data from RA patients have shown promising results for early and differential diagnosis, relapse prediction, extra-articular involvement, and response to specific treatments [115]. Given the high clinical heterogeneity of RA, the ability to identify responders and non-responders early is crucial for optimizing therapeutic strategies. In this context, integrating multi-omics data appears particularly promising, as it allows for a deeper biological characterization of different RA phenotypes beyond a purely clinical perspective [101]. Moreover, predictive models that combine clinical and biological data have highlighted the potential of AI in patient stratification and in predicting response to biological agents and tsDMARDs [95,98,100]. On the other hand, DL models applied to imaging have shown promising performance in the automated identification of synovitis, tenosynovitis, bone marrow edema, and structural joint damage [69,72]. These applications could contribute to greater standardization of image interpretation, reducing interobserver variability and improving diagnostic accuracy, especially in the early stages of the disease.

Surprisingly, in some cases AI models have proven superior to junior clinicians’ judgment in diagnosing rheumatic diseases, including RA [44,49,50,121,122]. These data are particularly robust for DL models and align with the results of a systematic review published in 2020 comparing AI with expert clinicians in medical imaging [123].

However, each computational paradigm presents specific limitations that should be considered when interpreting their results and evaluating their clinical applicability.

Traditional ML algorithms remain the most frequently adopted methods, particularly when structured clinical, laboratory, or biomarker data are available. These models generally require manual feature engineering and careful variable selection, making their performance strongly dependent on the quality and completeness of the input data. Furthermore, they may struggle to capture complex nonlinear relationships and high-dimensional interactions, although they often provide greater interpretability than DL models.

DL techniques have shown remarkable performance in imaging-based applications, including radiographs, ultrasound, and thermographic images. Nevertheless, their effectiveness largely depends on the availability of large, well-annotated datasets, which remain relatively scarce in rheumatology. In addition, deep neural networks are often regarded as “black-box” models, limiting the interpretability of their predictions and potentially reducing clinicians’ confidence in their adoption for routine practice [78]. In rheumatology, where therapeutic decisions often require a multidimensional and collaborative approach, understanding the biological and clinical rationale behind AI-generated predictions will be crucial to foster clinicians’ trust and integrate these tools into daily practice.

NLP has recently emerged as a promising tool for extracting information from clinical narratives, supporting medical documentation and assisting patient education. However, this model remains susceptible to hallucinations, inconsistent reasoning, and the propagation of outdated or inaccurate medical information. Its output also depends heavily on prompt formulation and the quality of the underlying training data, raising concerns regarding reliability, reproducibility, and regulatory compliance in clinical settings.

Other computational approaches, including simulation-based inference, graph-based methods, and network modeling, offer valuable opportunities to investigate disease mechanisms and biological pathways. However, these techniques are generally characterized by limited clinical validation, greater methodological complexity, and reduced accessibility for routine clinical implementation.

Finally, regardless of the computational methodology employed, most studies share common limitations, including relatively small sample sizes, retrospective study designs, single-center cohorts, heterogeneous datasets, and limited external validation. Many models are developed on relatively small, single-center, highly selected cohorts, leading to overfitting and limited generalizability [106]. Additionally, the heterogeneity of data collection protocols, diagnostic criteria, and clinical outcomes often makes it difficult to directly compare studies and externally validate proposed algorithms.

Another limitation involves the quality and standardization of data used to train models. For imaging, differences in MRI protocols, US image quality, and radiographic acquisition systems can significantly affect algorithm performance. Similarly, data from EMRs are often incomplete, fragmented, or characterized by high terminological variability, especially in free-text fields [107].

From a methodological perspective, many studies focus primarily on the statistical performance of algorithms, while evidence regarding the actual clinical impact of AI on patient outcomes remains limited. Few models have been evaluated in prospective settings or integrated into real-world clinical decision-making processes. Most evidence comes from retrospective or feasibility studies, highlighting the need for prospective clinical trials and large-scale multicenter validations.

These factors currently restrict the generalizability of many AI models and highlight the need for larger multicenter studies, standardized datasets, explainable AI strategies, and prospective clinical validation before widespread implementation in routine rheumatology practice.

Beyond methodological concerns, significant ethical and regulatory issues remain unresolved. Biases related to dataset selection, underrepresentation of specific populations, and potential inequalities in access to AI technologies could amplify existing disparities in healthcare. In this context, it will be essential to develop more transparent, inclusive, and validated models across ethnically and geographically diverse populations.

Despite these challenges, the future development of AI in RA appears highly promising. One innovative advancement could be the dynamic, longitudinal integration of data from various sources, including wearable devices, digital sensors, ePROs, and environmental data. Combining this information could enable continuous monitoring of disease activity and the creation of real-time predictive models capable of anticipating clinical exacerbations or loss of therapeutic response.

## 5. Future Perspectives

**Figure 3:**
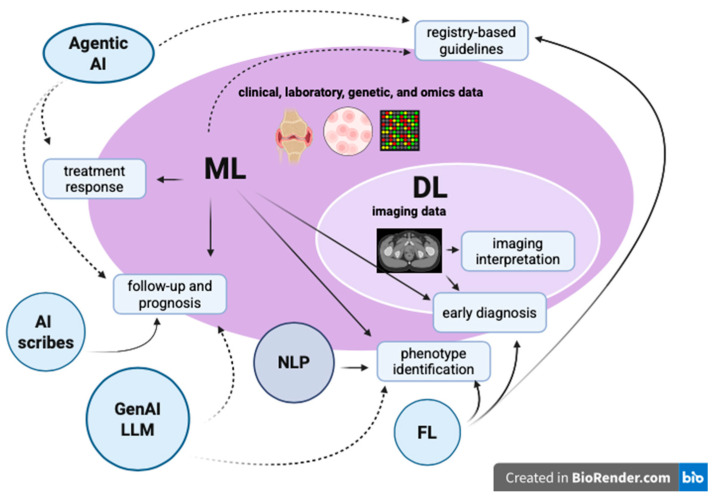
Current applications of AI models in RA domains and future perspectives. Abbreviations: AI: artificial intelligence; DL: deep learning; FL: Federated Learning; GenAI: generative artificial intelligence; LLM: large language models; ML: machine learning; NPL: natural language processing. The solid arrows indicate applications that have already been validated, while the dotted arrows indicate possible future areas of application. Created in Biorender. Rossella Talotta. (2026) (https://BioRender.com).

Despite the promising advantages of applying AI to RA, its implementation in clinical practice remains limited. The main obstacles include the use of small and poorly standardized datasets, frequent lack of external validation, and the predominance of single-center studies, all of which compromise data generalizability. Additionally, limited interpretability of models—particularly those based on DL—, difficulties in integrating them into clinical workflows, algorithmic bias, and regulatory issues constitute further barriers to large-scale adoption of AI in clinical practice [20]. However, the development of increasingly advanced models capable of merging clinical, imaging, biomarker, and omics data in multimodal approaches is growing exponentially. In this context, the use of longitudinal data will enable a better understanding of disease dynamics and the development of more accurate and personalized predictive models, as shown in Figure 3.

Here are the main future cornerstones for applying AI to improve the global management of patients with RA:

(1) AI may assist expert panels in formulating and continuously updating guidelines on crucial aspects of RA, ranging from early diagnosis to personalized treatments. In this context, it is important to note that applying AI models to the process of guideline creation—which mostly relies on the Consensus Development and GRADE methods—may result in more detailed and robust data review, drawing from registries, EHRs, and wearable devices. This approach can enhance objectivity, reduce bias, lower costs and time requirements, and enable continuous updates [124]. The growing availability of national real-world registries could promote the application of AI in drawing RA management guidelines from generalized real-world clinical data. Detailed analysis of registry data on recently introduced therapeutic molecules, such as JAKi, could better characterize their efficacy and safety profiles across multiple RA patient cohorts. For instance, data from the GISEA and ToRaRI studies may be analyzed to longitudinally assess efficacy, tolerability, and retention rates, which are essential for the development and validation of future predictive models [125,126]. However, AI models cannot completely replace human judgment, as expert supervision is required to draw final conclusions in the GRADE algorithm.
(2) Another area of great interest is the use of GenAI and large language models (LLMs) [33]. This category includes computational methodologies that do not fall within conventional ML paradigms, such as simulation-based inference, graph-based modeling, and network analysis. Compared to conventional predictive AI models, GenAI not only analyzes existing data or produces classifications but also generates new outputs based on learned patterns [127]. In biomedical research, GenAI can generate new hypotheses, simulate biological scenarios, identify synthetic combinations of biomarkers, and design de novo molecular structures with predefined therapeutic properties [127]. Therefore, these approaches are mainly employed to investigate biological mechanisms, disease pathways, or complex interactions that are difficult to capture using traditional predictive models. Although clinical applications in rheumatology are still in their early stages, GenAI could offer promising perspectives in the management of several diseases, including RA. Specifically, these technologies could potentially aid in identifying novel biomolecular signatures, simulating disease trajectories, integrating multimodal datasets, and generating hypotheses regarding therapeutic response or disease progression [33]. Furthermore, by leveraging omics and imaging data, generative models could support precision medicine approaches by identifying hidden biological patterns and informing the design of future therapeutic strategies. However, the ability to generate biologically plausible outputs does not necessarily ensure clinical validity, and issues such as hallucinations, data dependence, bias, and real-world validation remain significant limitations [127].
(3) An even more advanced and emerging field is Agentic AI. Agentic AI differs from conventional ML and static generative models because it operates as an autonomous decision-making agent, capable not only of making predictions but also of reasoning, planning, using external tools, maintaining contextual memory, and iteratively adjusting its decision-making process [127]. Although not yet validated for use in RA, Agentic AI could theoretically integrate real-time clinical, serological, imaging, genomic, and longitudinal data, continuously reassess disease activity, monitor therapeutic response, and support adaptive treat-to-target strategies through an iterative clinical decision-making process [127].
(4) The development of more robust, multicenter, and generalizable AI models, while addressing the ethical, regulatory, and logistical challenges of sharing sensitive data, could be enabled by Federated Learning (FL). FL is a validated algorithm that combines medical data from multiple centers to improve predictive accuracy and reduce the risk of bias associated with small or highly selected cohorts [128]. As such, FL is emerging as a relevant strategy in fields such as oncology, immunotherapy, and precision medicine [128]. In rheumatology, FL could address challenges associated with relatively small, single-center, and poorly heterogeneous datasets and enhance the robustness of predictive models for early diagnosis, prognostic stratification, and therapeutic response prediction while maintaining patient confidentiality. Looking ahead, FL could also enable the creation of international collaborative AI networks in rheumatology [128]. Data heterogeneity across centers, the need for protocol standardization, computational complexity, risks of distributed bias, and the establishment of appropriate regulatory and governance frameworks represent major obstacles to this approach.
(5) Finally, incorporating ambient AI scribes, which rely on LLMs, into daily clinical routines may reduce physician burnout and work exhaustion, which are largely due to the time spent on EHR documentation [129]. Ambient AI scribes enable the instantaneous transcription of conversations between physicians and patients to create a structured draft clinical note [130]. AI scribes are actively validated and increasingly adopted in rheumatology. The main limitations of this innovative approach are its costs and potential inaccuracies, which require constant human supervision.

## 6. Conclusions

Overall, AI is one of the most promising innovations in the management of RA. Although current evidence is still limited by methodological challenges and a lack of extensive clinical validation, recent progress suggests that AI could gradually transform the diagnostic, prognostic, and therapeutic approaches to the disease. The future development of more robust, interpretable, and clinically integrated models will be essential for translating the theoretical potential of AI into tangible benefits for patients with RA.

## Figures and Tables

**Table 1:** Classification criteria used for literature selection.

Domain	Category	Description
Data type	Clinical	Studies primarily based on clinical, laboratory, anamnestic, genetic, biomarker, questionnaire, EHR/EMR, or patient-reported outcome data, without direct use of medical imaging.
	Instrumental	Studies mainly using imaging or instrumental diagnostic data, including radiographs, MRI, ultrasound, thermography, HR-pQCT, scintigraphy, smartphone imaging, or other digital sensor-based modalities.
	Clinical + Instrumental	Studies integrating both clinical and instrumental/imaging data within the same analytical workflow or predictive model.
Task	Diagnostic	Studies focused on disease identification, classification, screening, early diagnosis, or severity assessment of RA and related rheumatic diseases using computational models.
	Predictive	Studies aimed at predicting disease risk, clinical outcomes, progression, remission, flare occurrence, or therapeutic response.
	Clinical Decision Support and Monitoring	Studies dedicated to clinical decision support, patient monitoring, workflow optimization, healthcare data management, or digital rehabilitation systems.
	Drug Discovery and Molecular Modeling	Studies involving biomarker discovery, therapeutic target identification, molecular mechanism analysis, drug repurposing, pharmacological screening, or computational biological simulations.
	Healthcare System and Infrastructure	Studies related to digital health platforms, datasets, telemedicine systems, healthcare infrastructures, methodological frameworks, or healthcare organization.
AI algorithm	Traditional Machine Learning	Conventional machine learning models, including Random Forest, support vector machine, XGBoost, logistic regression, k-nearest neighbors, Decision Trees, and classical ensemble methods.
	Deep Learning	Deep neural network architectures, including CNNs, DenseNet, ResNet, U-Net, transformer-based vision models, and other deep learning approaches.
	NLP/Generative AI	Natural language processing and generative AI methods, including text mining, knowledge extraction, BERT-based models, LLMs, and text-generative systems.
	Other Computational Approaches	Advanced computational methods not directly classifiable into previous categories, including graph-based modeling, simulation-based inference, fuzzy systems, optimization algorithms, and complex statistical-computational frameworks.

Abbreviations: CNNs: convolutional neural networks; EHR: electronic health record; EMR: electronic medical record; HR-pQCT: High-Resolution peripheral Quantitative Computed Tomography; LLMs: large language models; MRI: magnetic resonance imaging; RA: rheumatoid arthritis.

## Data Availability

No new data were created or analyzed in this study. Data sharing is not applicable to this article.

## Abbreviations

The following abbreviations are used in this manuscript:

ACPAs	anti-citrullinated peptide antibodies
AI	artificial intelligence
ANAs	antinuclear antibodies
APC	antigen-presenting cells
ASAS HI	assessment of spondyloarthritis international society health index
ASAS	assessment of spondyloarthritis international society
AUC	area under the ROC curve
AUROC	area under the receiver operating characteristic curve
axSpA	axial spondyloarthritis
BASDAI	Bath ankylosing spondylitis disease activity index
BDI	Beck depression inventory
bDMARDs	biologic disease-modifying anti-rheumatic drugs
BiLSTM	bidirectional long short-term memory
BMI	body mass index
BMO	bone marrow edema
CDU	color Doppler ultrasound
CNNs	Convolutional Neural Networks
CRP	C-reactive protein
csDMARDs	conventional synthetic disease-modifying anti-rheumatic drugs
CSF	cerebrospinal fluid
CTDs	connective tissue diseases
CTG-PAM	cell-tissue-graph-based pathological image analysis model
CV	cardiovascular; DL: deep learning
DAS28	disease activity score on 28 joints
DL	deep learning
DXA	dual-energy X-ray absorptiometry
EHR	electronic health record
EMR	electronic medical record
EMRS	electronic medical records system
ePROS	electronic patient-reported outcomes
ESR	erythrocyte sedimentation rate
EULAR	European League Against Rheumatism
FL	Federate Learning
FLSs	fibroblast-like synoviocytes
FNN	Feed-forward neural network
GCA	giant cell arteritis
GenAI	generative artificial intelligence
GEO	Gene Expression Omnibus
GIMS	gout intelligent management system
GMLVQ	generalized matrix relevance
HLA	allele human leukocyte antigen
HR-pQCT	High-Resolution peripheral Quantitative Computed Tomography
HRQOL	health-related quality of life; IIM: idiopathic inflammatory myopathy
IL	interleukin-1
ILD	interstitial lung disease
KD	Kawasaki disease; KLG: Kellgren–Lawrence grade
JAKi	Janus kinase inhibitors
LASSO	least absolute shrinkage and selection operator
LDA	low disease activity
LLMs	large language models
LN	lupus nephritis
MCTD	mixed connective tissue disease
ML	machine learning
MLS	macrophage-like synoviocytes
MMDLS	multimodal DL system
MMPs	matrix metalloproteinases
MRI	magnetic resonance imaging
NLP	natural language processing
NLR	neutrophil-to-lymphocyte ratio
NPSLE	neuropsychiatric systemic lupus erythematosus
NSAIDs	non-steroidal anti-inflammatory drugs
OA	osteoarthritis
OESS	OMERACT–EULAR Synovitis Scoring system
OP	osteoporosis
PBMCs	peripheral blood mononuclear cells
PD	power Doppler
PLR	platelet-to-lymphocyte ratio
PROs	patient-reported outcomes
PSA	psoriatic arthritis
PVNS	pigmented villonodular synovitis
RA	rheumatoid arthritis
RAPID-3	Routine Assessment of Patient Index Data 3
RCT	randomized controlled trial
RF	rheumatoid factor
RMD	rheumatic musculoskeletal disease
SEC	secukinumab
SHAP	Shapley additive explanation
SLE	systemic lupus erythematosus
SNP	single nucleotide polymorphism
SpA	spondyloarthritis
SS	Sjögren’s syndrome
SSc	systemic sclerosis
TEM	transmission electron microscopy
TCZ	tocilizumab
Th	T helper
TJC	tender joint count
TNFi	tumor necrosis factor inhibitors
TNF-α	tumor necrosis factor α
tsDMARDs	targeted synthetic disease-modifying anti-rheumatic drugs
U-Net	U-shaped convolutional neural networks
US	ultrasound
WOMAC	Western Ontario and McMaster Universities Arthritis Index
XAI	explainable artificial intelligence
XGBoost	extreme gradient boosting
